# Nonlinear Stochastic Equation within an Itô Prescription for Modelling of Financial Market

**DOI:** 10.3390/e21050530

**Published:** 2019-05-25

**Authors:** Leonardo S. Lima

**Affiliations:** Departamento de Física, Centro Federal de Educação Tecnológica de Minas Gerais, Belo Horizonte, MG 30510-000, Brazil; lslima7@yahoo.com.br

**Keywords:** price dynamics, stochastic differential equation, Itô calculus

## Abstract

The stochastic nonlinear model based on Itô diffusion is proposed as a mathematical model for price dynamics of financial markets. We study this model with relation to concrete stylised facts about financial markets. We investigate the behavior of the long tail distribution of the volatilities and verify the inverse power law behavior which is obeyed for some financial markets. Furthermore, we obtain the behavior of the long range memory and obtain that it follows to a distinct behavior of other stochastic models that are used as models for the finances. Furthermore, we have made an analysis by using Fokker–Planck equation independent on time with the aim of obtaining the cumulative probability distribution of volatilities P(g), however, the probability density found does not exhibit the cubic inverse law.

## 1. Introduction

In general, physics has long been a large source of ideas for economics. Every investor would like to be able to predict the price of a stock in the same way as physicists predict the trajectory or position in function of time of a particle. Since the prices of stocks of companies exhibit unpredictable fluctuations, they can be modeled by stochastic differential equations which becomes very important in the pricing of financial derivatives [1,2]. Since the celebre equation for price dynamics of the European market derived by Black and Scholes [3] until nowadays, the modeling of financial has been focused to simulate the behavior of market structure, trading mechanism and price dynamics [4]. Mike and Farmer [5] have made an empirical behavioral model to simulate the dynamic of stock price formation. In the following, Gu and Zhou [6] have modified the MF model by incorporating long memory into aggressiveness of incoming orders [7].

A very important model in modelling of financial market is the Ising model, $H=\sum_{\langle ij\rangle }J_{ij}\sigma_{i}\sigma_{j}$. It constitutes in a very general class of stochastic dynamical model developed to describe interacting elements, particles, and agents in physics and biology. The tendency towards imitation is governed by $J_{ij}$, which is the “coupling strength”. This means the tendency towards noisy behavior is governed by $\sigma_{i}$ of the noise term. Hence, the value of *J* relative to σ determines the outcome of the battle between order and disorder, and eventually the structure the market prices [8]. We consider the price $p_{i}$ as analogous to the spins $\sigma_{i}^{z}$ of Ising chain and the magnetization interpreted as volatility, which is the modulus of the return, g=|r(t)| [9,10,11,12,13,14,15,16,17,18,19]. The study of volatility is crucial to reveal the underlined mechanism of markets dynamics, and it is also useful for traders because it helps them in estimating risk and optimization of portfolio [20]. Another quantity of interest in price dynamics is the return r(t). Its statistical analysis is well known as distribution of returns: $r\left(t\right)\approx S\left(t\right)=\ln\left(X\left(t+\Delta t\right)\right)-\ln\left(X(t)\right)$, where the long tail cumulative probabilities distribution of volatilities *g* obeys to an inverse cubic-law $P\left(g\right)∼g^{-\gamma }$, where γ∼3 is the tail exponent [4,6,9,14,21,22,23,24,25,26,27].

The modelling of stocks throughout stochastic differential equations is also often employed in the modelling of market [28,29,30,31,32,33,34]. Furthermore, modelling through a set of linear equations [35] and non-linear equations [36] is also often employed. Since exponentials and Gaussian functions can emerge in economics theories [37,38,39], it is well known that such functions can be generalized into a nonlinear one: $f\left(x\right)=e_{q}^{ax}$, where $e_{q}$ is the *q*-exponential function defined in non-extensive statistical mechanics [39] as $e_{q}^{x}\equiv {\left[1+(1-q)x\right]}_{+}^{1/(1-q)}$, ($e_{1}^{x}=e^{x}$), with ${\left[1+(1-q)x\right]}_{+}=1+\left(1-q\right)x$ if z>0 and zero otherwise. An important thing related to finances is the observation of scaling laws exhibited by large price fluctuations, being corroborated for practically all types of financial data and markets [40,41,42,43].

In this paper, we investigate an Itô diffusion model with additive noise and nonlinear terms as a possible model for the financial market. The aim is to determine if the model obeys stylised facts about financial markets such as the exponent of the long tail distribution of volatilities as well as the long range memory or Hurst index. The stochastic differential equation with nonlinear terms until quadratic terms has been already proposed as a model for stock market fluctuations and crashes, being known as Bouchaud-Cont Langevin model [44]. Here, we study a more general model with terms of higher order. The case of the cubic potential and higher order with additive white noise has been treated in [28,31]. The case of multiplicative noise has been treated in [29]. The plan of this paper is the following. In Section 2 we discuss the economic entropy. In Section 3, we describe the stochastic model. In Section 4, we present the numerical results. In Section 5, we make a mathematical analysis using the Chapman–Kolmogorov equation. In Section 5, we present our conclusions and final remarks.

## 2. Economic Entropy

When we consider the details of microstructure of an economic system, one can understand the economic entropy. In the economy the entropy gives similarly a measure of the total number of available ’economic’ states, whereas the energy measures the probability that any state in the ’economic phase state’ will be realised. In a system of traders, it could be the total number of ways that the money can be distributed across the agents [1]. In macroeconomics, the entropy function *S* takes the role of a production function of the macroscopic economic system. In microeconomics, the entropy is used to find the optimal number of different professionals in a company, the best choice of stocks in a portfolio, and so on. By considering the total number of elements $N=N_{1}+N_{2}$ of a binary system with two different categories, containing $N_{1}$ and $N_{2}$ elements of two types. One has the binary entropy or utility function

(1) $$S\left(N_{1},N_{2}\right)=N\ln N-N_{1}\ln N_{1}-N_{2}\ln N_{2}.$$

One can rewrite it making $x=N_{1}/N$ and hence $1-x=N_{2}/N$ to obtain

(2) $$S\left(x\right)=-N\left[x\ln(x)+(1-x)\ln(1-x)\right].$$

For a company with two different types of commodities, we have the Cobb-Douglas utility function $F=N_{1}^{\varepsilon }N_{2}^{1-\varepsilon }$, where the exponent ε∈[0.5,0.7] The “elasticity” parameter ε allows for adaptation of the function to data while *S* emerges from an entropy theoretical framework containing no free parameter and thus has true predictive power.

## 3. Phenomenological Itô Equation

The model of interest is defined by

(3) $$dX\left(t\right)=\left[\alpha X\left(t\right)-\mu {\left(X\left(t\right)\right)}^{3}-\delta {\left(X\left(t\right)\right)}^{6}\right]dt+\beta \left(X\left(t\right),t\right)dW\left(t\right),$$

where W(t) is a Winner process. X(t)=p(t) is the price of a derivative. The equation above can be used to describe the behavior of a particle in Brownian motion under action of an potential of type $\phi_{6}$ in which the prices of stocks ought obey. In the absence of sixth order term, the potential reduction to one of double well separated by a barrier of potential of height $V_{0}$. When we include the sixth order term, one have more wells. As in the neighboring of each minimum, the prices tend to have an oscillatory behavior, the presence of a noise can cause the prices to move from a well to another well, reflecting a crash in the market [44]. Moreover, we have a dissipative force given by $-\gamma \dot{x}$ represented by the friction term in Langevin’s equation and an environment stochastic white noise ζ(t), which if relates with the Winner process W(t) by

(4) $$W\left(t\right)=\int_{t_{0}}^{t}\zeta \left(t^{\prime }\right)dt^{\prime }.$$

Although W(t) be the integral of ζ(t), the inverse is not true, i.e., ζ(t)≠dW(t)/dt, since

(5) $$\frac{dW(t)}{dt}=\lim_{\Delta t\to 0}\frac{\Delta W(\Delta t)}{\Delta t}∼\frac{1}{\sqrt{\Delta t}}\to \infty .$$

W(t) is a Markovian process with normal probability distribution. In a general way, non linear terms in Equation (3) must model situations of instabilities with the appearance of crashes, where “panic” is self reinforcing. They can also be responsible for the sudden collapse of speculative bubbles [44].

## 4. Numerical Results

**Figure 1:**
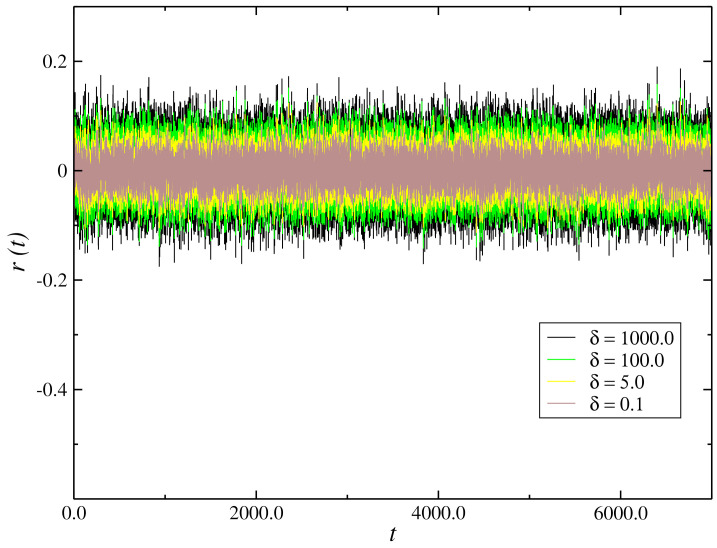
Time evolution of the return r(t)≈S(t)=ln(X(t+Δt))−ln(X(t)) for values of parameters $\beta =1.0\times 10^{3}$ and α=1.0 and different values of δ.

We perform a simulation of the model Equation (3) with the term βW(t) being the Winner increment and an additive white noise of standard deviation $\sigma_{w}=\sqrt{\Delta t}$. One can write the Winner increment as $\beta dW\left(t\right)∼\sqrt{dt}\beta R_{G}$, where $R_{G}$ is an aleatory generator number with a Gaussian distribution of mean zero and variance $\sigma_{w}^{2}=1$. The dynamic behavior of the return r(t)≈S(t)=ln(X(t+Δt))−ln(X(t)) is shown in Figure 1 for parameter values $\beta =1.0\times 10^{3}$, α=1.0 and different values of δ. We have the time series of changing of prices oscillating quickly within a range. It suffers a large change with the increase of the coupling $x^{6}$ as shown in the Figure 1.

**Figure 2:**
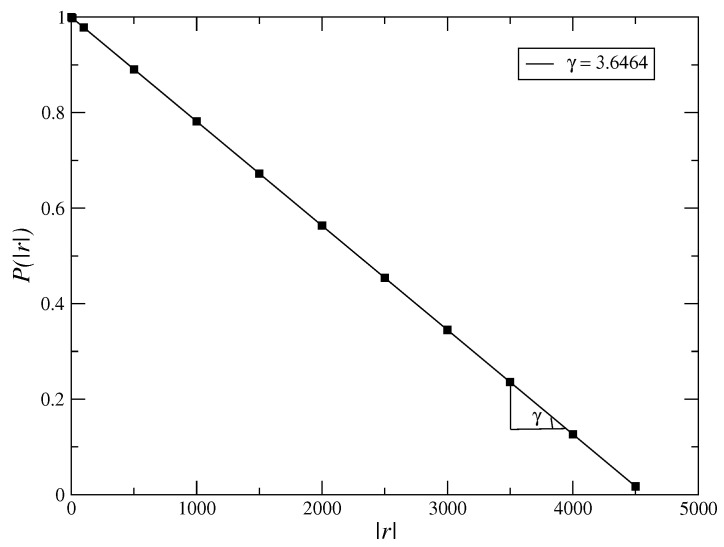
Long tail distribution of the volatilities, g=|r|, P(|r|) for values β=1000.0, α=1.0, μ=1.0 and δ=1000.0. The least-squares fits of power laws varies as $f\left(|r|\right)∼{\left|r\right|}^{-\gamma }$ for the long tail distribution, where we found the tail index given by γ∼3.65.

**Figure 3:**
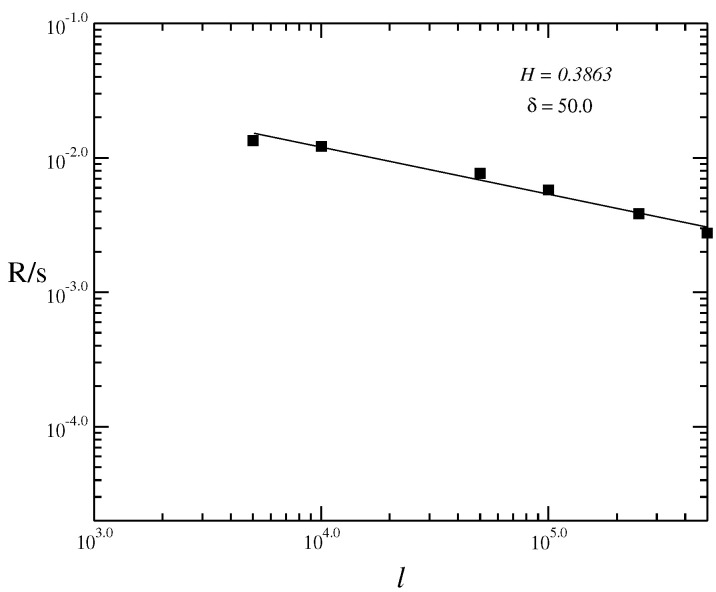
Log-Log graphic using the rescaled range method (RS) of the volatility $\log\left(R/s\right)$ vs. t=logn for a time series, determined for the value of β=1000.0, α=1.0 and δ=50.0. The Hurst indexes is obtained as H=0.386(3).

**Figure 4:**
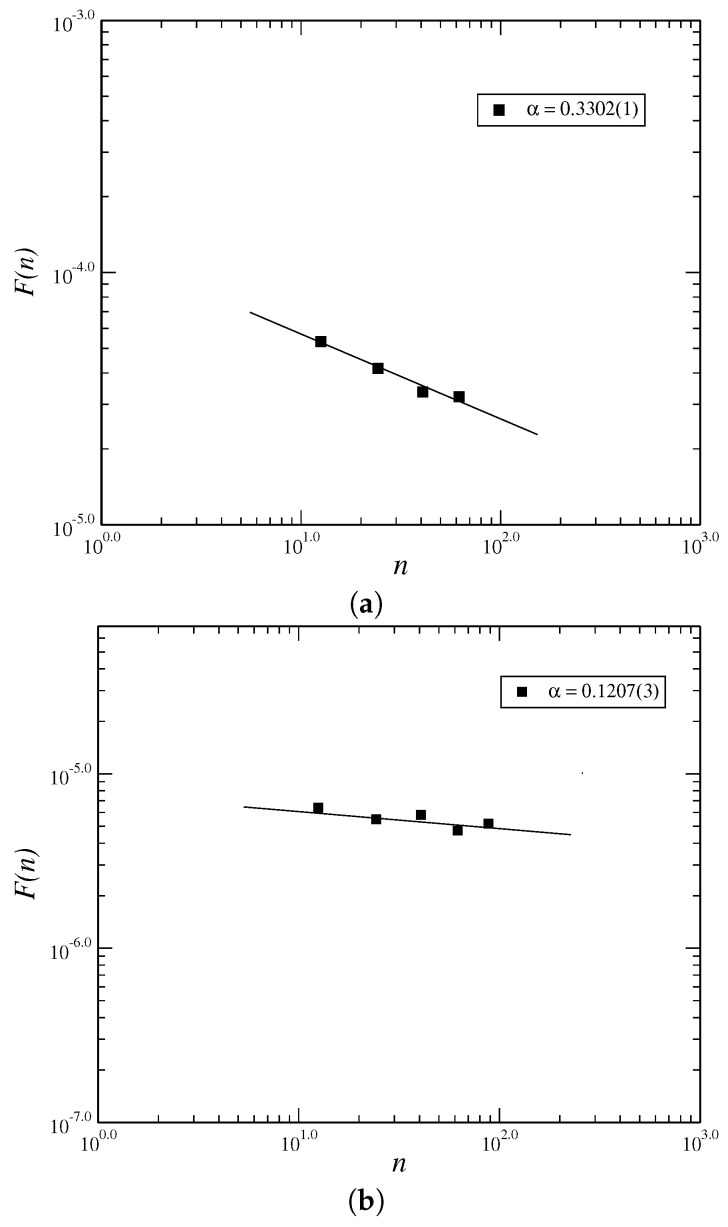
Log-Log graphic using Detrended Fluctuation Analysis (DFA) method for $\log\left(F(n)\right)$ vs. logn for the returns (**a**) and volatilities (**b**) respectively, determined for the value of β=1000.0, α=1.0 and δ=50.0.

In Figure 2, we analyse the behavior of the long tail distribution of the volatilities g=|r(t)|, P(g) which must obey the power law $P\left(g\right)=g^{-\gamma }$, with γ∼3 [21,22,23,24]. As we obtain the value of exponent of long tail of the fitted curve near to 3 (γ∼3.65), hence, we obtain that the model must reproduce to well stylized facts of the financial market. However, it is necessary to analyse the Hurst index, being the addition of more terms, such as $\phi^{8}$ terms, must generate an increase of it, making the exponent of the long tail distribution nearer 3. However, the high order terms in Equation (3) are not smaller than inferior order terms due to the fact the Taylor expansion is not valid in this case, i.e., the usual approach $f\left(x+h\right)=f\left(x\right)+\frac{df}{dx}\cdot h+r\left(h\right)$, with $\lim_{h\to 0}\frac{r(h)}{\left|h\right|}=0$ not if it applies in this case. In a general way, there have been many models employing different mathematical methods to describe the dynamical behavior of financial markets with the objective of seeking general evidences for their behavior. However, few model are suitable. Moreover, there have been many kinds of assets in the financial markets, such as stocks, futures, and other financial tools, each of them having different price behaviors. Higher-order terms in general force to non Gaussian distributions in Equation (3) since they still exhibit an exponential dependence that is not verified by most of the financial markets. In Figure 3, we calculate the Hurst index using the Rescaled Range method (RS). We plot the graphic of the volatility log(R/s) vs. t=logn for the time series, for values of parameters β=1000.0, α=1.0, μ=1.0 and δ=50.0. In the RS method, we have that *R* is the range and *s* is the standard deviation. We estimate the Hurst index using the RS method given by H∼0.39. The RS method is an older method that is not used very often nowadays. The Detrended Fluctuation Analysis (DFA) is recently more employed to analyse the behavior of time series. As the value of Hurst index employing the RS method is within the range H∈(0.0,0.5] we have a time series with long-term switching between high and low values in adjacent pairs, meaning that a single high value will be followed by a low value [45]. Employing the DFA method, we have obtained the exponent α as α∼0.33 for the returns and α∼0.12 for the volatilities (Figure 4). The exponent obtained using the DFA method is similar to the Hurst exponent, except that DFA may also be applied to signals whose underlying statistics (such as mean and variance) or dynamics are not stationary or if they relate to measure based upon spectral techniques such as autocorrelation and Fourier transform. Thus, we have obtained that the inclusion of higher order terms, such as sixth order terms on the potential $\phi^{4}$, Equation (3), has improved the model making that the model tends to a possible model of the market, since it has made the exponent of the long-tail cumulative probability distribution nearer to 3 and thus, tends to obey the inverse cubic law [28].

## 5. Analysis by Fokker–Planck Equation

Equation (3), which in the linear, deterministic case (that is, for a potential given by αX and W=0) reduces to the classical Fokker–Planck equation, describing the particle transport dynamics (price dynamics) in disordered media driven by highly irregular or stochastic field forces [46].

When the transition probabilities are zero, the differential Chapman–Kolmogorov equation reduces to Fokker–Planck equation [33]. From stochastic equation Equation (3), we obtain the time development of an arbitrary f(X(t)) using the Itô formula [33]

(6) $$\begin{array}{cc} f\left[X(t)+dX(t)\right]-f\left[X(t)\right] & =f^{\prime }[\left(X\left(t\right)\right]\left[[\alpha X\left(t^{\prime }\right)-\mu {\left(X\left(t^{\prime }\right)\right)}^{3}-\delta {\left(X\left(t^{\prime }\right)\right)}^{6}]dt+\beta dW\right]\\ & +\frac{\beta^{2}}{2}f^{\prime \prime }\left[X\left(t\right)\right]{\left(dW\right)}^{2}, \end{array}$$

where higher order terms have been discarded, and ${\left(dW\left(t\right)\right)}^{2}=dt$. Taking the average of both sides in the equation above and defining $\gamma =\beta^{2}$, we get

(7) $$\langle \frac{df}{dt}\rangle =\langle \left[\frac{\partial f}{\partial x}\left[\alpha X\left(t^{\prime }\right)-\mu {\left(X\left(t^{\prime }\right)\right)}^{3}-\delta {\left(X\left(t^{\prime }\right)\right)}^{6}\right]+\frac{\gamma }{2}\frac{\partial^{2}f}{\partial x^{2}}\right]\rangle .$$

Using

(8) $$\begin{array}{cc} \frac{d}{dt}\langle f(X\left(t\right)\rangle & =\frac{d}{dt}\int_{-\infty }^{\infty }dxf\left(x\right)P\left(x,t\right)=\int_{-\infty }^{\infty }dxf\left(x\right)\frac{\partial }{\partial t}\left[P(x,t)\right]\\ & =\int_{-\infty }^{\infty }\frac{\partial f}{\partial x}\left[\alpha X\left(t^{\prime }\right)-\mu {\left(X\left(t^{\prime }\right)\right)}^{3}-\delta {\left(X\left(t^{\prime }\right)\right)}^{6}\right]P\left(x,t\right)dx\\ & +\frac{\gamma }{2}\int_{-\infty }^{\infty }\frac{\partial^{2}f}{\partial x^{2}}P\left(x,t\right)dx \end{array}$$

we integrate by parts and discard surface terms to obtain

(9) $$\begin{array}{cc} \int_{-\infty }^{\infty }dxf\left(x\right)\frac{\partial }{\partial t}\left[P(x,t)\right] & =\int_{-\infty }^{\infty }f\left(x\right)\frac{\partial }{\partial x}\left\{\left[\alpha X\left(t^{\prime }\right)-\mu {\left(X\left(t^{\prime }\right)\right)}^{3}-\delta {\left(X\left(t^{\prime }\right)\right)}^{6}\right]P\left(x,t\right)\right\}dx\\ & +\frac{\gamma }{2}\int_{-\infty }^{\infty }f\left(x\right)\frac{\partial^{2}}{\partial x^{2}}\left[P(x,t)\right]dx. \end{array}$$

and hence

(10) $$\frac{\partial }{\partial t}P\left(x,t\right)=-\frac{\partial }{\partial x}\left\{\left[\alpha X\left(t^{\prime }\right)-\mu {\left(X\left(t^{\prime }\right)\right)}^{3}-\delta {\left(X\left(t^{\prime }\right)\right)}^{6}\right]P\left(x,t\right)\right\}+\frac{\gamma }{2}\frac{\partial^{2}}{\partial x^{2}}P\left(x,t\right).$$

The associated Fokker–Planck equation to the above model is given by

(11) $$\;\frac{\partial P(x,t)}{\partial t}=\frac{\partial [\left(-\alpha x+\mu x^{3}+\delta x^{6}\right)P\left(x,t\right)]}{\partial x}+\frac{\gamma }{2}\frac{\partial^{2}\left[P\left(x,t\right)\right]}{\partial x^{2}}.$$

Taking the Fourier transform of the Fokker–Planck equation, provided we can simultaneously guarantee the normalization of the probability density and in which P(x) is reasonably well behaved, we take the boundaries at infinity for P(x,t) as $\lim_{x\to \infty }P\left(x,t\right)=0$ and therefore $\partial_{x}P\left(x\right)$ being reasonably well behaved. As $\lim_{x\to \infty }\partial_{x}P\left(x,t\right)=0$ thus, a nonzero current of probability at infinity will usually require that the terms in the equation above become infinite there [33]. We use the initial condition $P\left(x=x_{0},t=0\right)=P_{0}$. The Fokker-Plank equation above, independent on time, can be analytically solved making the power series expansion

$P\left(x\right)=\sum_{n=-\infty }^{\infty }a_{n}x^{n}$ generating the following recurrence relations

(12) $$\begin{array}{c} \left(m+2\right)\left(m+1\right)a_{m+2}-\frac{2\alpha m}{\beta }a_{m}-\frac{2(\alpha +k)}{\gamma }a_{m}=0\\ \mu \left(m-2\right)a_{m-2}-\frac{6\mu }{\gamma }a_{m-2}=0\\ \delta \left(m-5\right)a_{m-5}-\frac{12\delta }{\gamma }a_{m-5}=0. \end{array}$$

We have for 0≤m≤2

(13) $$a_{m}=\frac{\beta (m+2)(m+1)}{2\alpha (m+1)+2k}a_{m+2}.$$

Hence, we obtain P(x) as

(14) $$P\left(x\right)=\left[1+\frac{\gamma }{2}\left(\alpha +k\right)x^{2}+\frac{1}{3\beta^{2}}\left(\alpha +k\right)\left(3\alpha +k\right)x^{4}+\cdots \right]a_{0}+\left[x+\frac{1}{3\gamma }\left(2\alpha +k\right)x^{3}\cdots \right]a_{1}.$$

For 2≤m<5, we have $a_{m-2}=0$ and for m≥5, we also get $a_{m-5}=0$. Therefore,

(15) $$P\left(x\right)=a_{0}+a_{1}x,$$

where the constants $a_{0}$ and $a_{1}$ are determined by the initial conditions, being $a_{0}=P_{0}$ and *k* the separation constant in Equation (11). For m≤−1, we have $a_{m}=0$ for all *m*. From the normalization condition, the second term in the density probability above must be zero and therefore, all coefficients $a_{1}$ must cancel. Therefore, we have

(16) $$P\left(x,t\right)=P_{0}e^{-kt}$$

To ensure the normalization of the probability density, $P_{0}$ must be non zero only within interval −ε≤x≤ε and zero out it. Consequently, we have $P\left(x,t\right)=\left(1/2\varepsilon \right)e^{-kt}$.

The cumulative probability density F(x) for the probability density above in the limit of *x* large is given by

(17) $$F\left(x,t\right)=\int_{-\infty }^{x}P\left(x^{\prime }\right)dx^{\prime }=\frac{1}{2\varepsilon }\left(x-\varepsilon \right)e^{-kt}≃\frac{1}{2\varepsilon }xe^{-kt}$$

Despite the exponential decreasing of time behavior for the cumulative probability, we have the cumulative probability distribution of the volatilities P(|r(t)|) that must exhibit the cubic inverse law behavior, $P\left(|r|\right)=1/{\left|r\right|}^{3}$, for the long tail, and not the cumulative probability density standard. For μ, δ=0, the Equation (11) can be easily solved giving $\nu \left(t\right)=x_{0}e^{-i\alpha t}$ and $\kappa \left(t\right)=\frac{\gamma }{2\alpha }\left(1-e^{-2\alpha t}\right)$ and hence, we have a Gaussian distribution for P(x,t) given by

(18) $$P\left(x,t\right)=\frac{1}{\sqrt{2\pi \kappa (t)}}e^{-\frac{{\left(x-\nu \left(t\right)\right)}^{2}}{2\kappa (t)}},$$

were in t→∞ limit, we have P(x,t)=P(x) given by

(19) $$P\left(x\right)=\sqrt{\frac{\alpha }{\pi \gamma }}e^{-\frac{\alpha x^{2}}{\gamma }}.$$

Therefore, the probability density is non-Gaussian when terms of type $\phi^{4}$ and $\phi^{6}$ and higher order are included in the model.

## 6. Conclusions

In summary, we have investigated a generalized nonlinear Itô’s stochastic model or generalized Itô diffusion model as a mathematical model for the price dynamics of the financial market. As the $\phi_{n}$ has local minimums for $u=u_{0}$ and maximum for u=u∗, beyond which the potential plummets to −∞, there are height barriers separating the stable from unstable regions. Near to local minimum, the particle has a random harmonic-like until an activated event driven by noise term brings the particle near u∗. In financial terms, the regime where the particle oscillates around $u=u_{0}$ is the random walk regime. This normal regime can be interrupted by “crashes”, where the derivative of the price becomes large and negative due to the risk aversion given μ, δ terms which enhance the drop in price. We have calculated the Hurst index using both the RS and DFA methods and verified the behavior of the long tail distributions of the volatilities that must obey to the inverse cubic law behavior [21,22,23,24]. Our results show that the inclusion of higher order terms in the Bouchaud-Cont Langevin model [44] and on models of Refs. [28,29,30,31] still make it so that they are obeyed by the financial markets. In a general way, there have been many methods, employing mathematical models, to describe the price dynamics in finance, seeking general evidences. However, there are few models suitable for real situations. Furthermore, there are many kinds of assets in the financial markets, such as stocks, futures, and other financial tools, each of them with different price behaviors.

In a general way, interaction terms of the form $\lambda_{4}\phi^{n}/n!$ can be represented by connected graphs corresponding to given initial and final states with *n* lines meeting at each vertex. Interaction terms proportional $\phi^{6}$ introduced in the $\lambda_{4}\phi^{4}$ theory can give a further contribution to the six-point amplitude and we could choose $\lambda_{6}\phi^{6}$ so that it cancels the divergence terms that are left. Therefore, we could adjust nonlinear terms in the Equation (3) with the aim to give an empirical evidence on the relationships between trading volume and return volatility of the Bitcoin market [47].
